# RACIAL COMPARISONS OF RECEPTIVITY TO A BLOOD-BASED BIOMARKER TEST FOR ALZHEIMER RISK

**DOI:** 10.1093/geroni/igad104.3547

**Published:** 2023-12-21

**Authors:** Eleanor Skemp, Hyun Jin Cho, Brian Carpenter

**Affiliations:** Washington University in St. Louis, St. Louis, Missouri, United States; Washington University in St. Louis, St. Louis, Missouri, United States; Washington University in St. Louis, St. Louis, Missouri, United States

## Abstract

Blood-based biomarker (BBB) tests to assess risk for developing Alzheimer disease (AD) have recently been approved for clinical use. Little is known about people’s interest and concerns about these new tests, although the relative simplicity of the tests may help reduce disparities in early diagnosis for AD in historically marginalized groups, such as African Americans. The current report uses data from a national survey of adults in the United States (N = 1,274) conducted in 2022, oversampling African Americans. In addition to demographic questions, respondents rated the importance of different factors they would weigh when deciding whether to take a BBB test and their trust with the healthcare system. The sample was diverse in age (M = 48.6, SD = 17.6, range = 18-91), balanced in gender (55.3% female) and race (48.1% African American), and ranging in education (41.1% < BA). A higher percentage of respondents rated the accuracy (69.2%) and usefulness (64.5%) of the test as very important to their decision, compared to cost (40.8%) and convenience (38.0%). However, African Americans rated cost, convenience, confidentiality, and the potential for learning about other medical problems as more important than White respondents (chi-square p’s < .01). Contrary to hypotheses, trust in the healthcare system was not significantly related to decisional factors. Results from this study have implications for how to tailor culturally sensitive messages about new tests for the early detection of dementia risk.

